# Association between genetic polymorphisms of cytochrome P450 2C19 and the risk of cerebral ischemic stroke in Chinese

**DOI:** 10.1186/1471-2350-15-83

**Published:** 2014-07-17

**Authors:** Shuzhen Gu, Yan Sun, Ruifa Han, Lin Wang, Dongliang Wang, Jizuo Wang, Xin Li

**Affiliations:** 1 Department of Neurology, the Second Hospital of Tianjin Medical University, Tianjin 300211, China; 2 Department of Electromyography, Luoyang Central Hospital affiliated to Zhengzhou University, Luoyang 471009, China; 3 Tianjin Institute of Urology, the Second Hospital of Tianjin Medical University, Tianjin 300211, China; 4 Department of Gerontology, the Second Hospital of Tianjin Medical University, Tianjin 300211, China

**Keywords:** Cerebral ischemic stroke, CYP2C19, Genetic polymorphism

## Abstract

**Background:**

Cytochrome P450 (CYP) 2C19 is a very important drug metabolizing enzyme. Although the single nucleotide polymorphisms (SNPs) of CYP2C19 G681A and G636A have been suggested that they may increase the incidence of cardiovascular events, the relationship between SNPs in CYP2C19 and cerebral ischemic stroke (CIS) are unclear. The aim of this study was to investigate the correlation between the distribution of G681A and G636A polymorphisms in CYP2C19 gene and the risk of CIS in Chinese.

**Methods:**

The peripheral blood DNA was extracted from 299 patients with CIS and 295 healthy controls. The genotyping was conducted using the polymerase chain reaction-restriction fragment length polymorphism. The sampled sequencing was applied to verify the correctness of genotyping results. Both the genotype and allele distributions were compared in patients with CIS and healthy controls.

**Results:**

The frequencies of CYP2C19 681AA (11.7% vs. 2.7%; *P* = 0.000), 636AA (4.0% vs. 0.7%; *P* = 0.007), 636AG (7.0% vs. 2.2%; *P* = 0.038) genotype, CYP2C19 681A (30.9% vs. 20.8%; *P* = 0.000) and 636A (13.0% vs. 5.8%; *P* = 0.000) allele in the CIS group are significantly higher than those in the controls. The frequencies of CYP2C19 681AA (16.7% vs. 8.6%; *P* = 0.036), CYP2C19 636AA (7.0% vs. 2.2%; *P* = 0.038) genotype, CYP2C19 681A (36.4% vs. 27.6%; *P* = 0.023) and CYP2C19 636A (17.5% vs.10.3%; *P* = 0.010) allele in the recurrent stroke group are significantly higher than those in the first onset group. Multivariate logistic regression analysis of risk factors for cerebral ischemic stroke and recurrent stroke respectively suggests that the CYP2C19 681AA genotype may be an independent risk factor for CIS (OR = 6.179, 95% CI: 2.285 ~ 16.708; *P* = 0.000) and recurrent stroke (OR = 2.305, 95% CI: 1.121 ~ 4.743; *P* = 0.023).

**Conclusions:**

The AA genotype and A allele of CYP2C19 G681A may be related to the occurrence and recurrence of cerebral ischemic stroke.

## Background

The occurrence of cerebral ischemic stroke is influenced by eating habits, environment, genetic factors and so on. Fundamentally, genetic factors involve in a series of key enzymes and receptors on many metabolic pathways. Therefore, the study of the genetic risk factor for cerebral ischemic stroke has become a hot spot currently.

Cytochrome P450(CYP) 2C19 is a very important drug metabolizing enzyme, which involves in approximately 2% of the clinical drug metabolism. The activity of CYP2C19 enzyme not only exhibits a significant ethnic heterogeneity, but also has an obvious differences between individuals. It is believed that the differences are mainly caused by genetic variations. Many researchers have investigated the molecular mechanism of CYP2C19 enzyme polymorphisms, which include the wild-type CYP2C19*1, CYP2C19*2, CYP2C19*3, CYP2C19*4, CYP2C19*5, CYP2C19*6, CYP2C19*7, CYP2C19*8, etc. CYP2C19*2 and CYP2C19*3 are the main variants in CYP2C19, while others are relatively rare in humans.

Studies have shown that the single nucleotide polymorphisms (SNPs) of CYP2C19*2(CYP2C19 G681A, rs4244285) and CYP2C19*3(CYP2C19 G636A, rs4986893) may increase the incidence of cardiovascular events [1-3]. Whereas there are less research about the relationship between SNPs in CYP2C19 and cerebral ischemic stroke. So we carried out a case-control study to investigate the relationship between the distribution of G681A and G636A polymorphisms in CYP2C19 gene and cerebral ischemic stroke in Chinese Han population.

## Methods

### Study participants

From May 2011 to March 2013, we recruited 299 patients with acute cerebral ischemic stroke who were admitted to the Neurology Department of the second hospital, Tianjin Medical University, Tianjin, China. All patients were diagnosed as acute cerebral ischemic stroke, which was diagnosed by a neurologist according to the diagnostic criteria determined by the guidelines for the primary prevention of stroke: a guideline for healthcare professionals from the American Heart Association/American Stroke Association [4] and confirmed by computed tomographic (CT) scan and/or conventional magnetic resonance imaging (MRI) of the brain; the time from incidence to hospitalization was less than seven days. We excluded patients who had cerebral hemorrhage, transient ischemic attack (TIA), cerebral venous thrombosis, nervous system infection, neurodegenerative disease, hepatic and renal dysfunction, thrombocytopenia and tumor. In addition, patients were excluded when they took PPI, tricyclic antidepressants, antiepileptics and antipsychotics.

Controls enrolled in this study may have high blood pressure, diabetes, smoking and other vascular risk factors, excluding history of cerebral ischemic stroke, TIA, atrial fibrillation, myocardial infarction and venous thrombosis.

All subjects were unrelated Chinese Han. There were no significant differences in the age and sex distributions. Informed consent was obtained from all subjects, and the study was approved by the Ethical Committee of Tianjin Medical University.

### Data collection

All subjects underwent a comprehensive medical history, physical examination and clinical chemistry analysis before enrollment. Diagnosis of hyperlipidemia was based on China’s adult dyslipidemia prevention guide [5]. Hypertension was defined as a blood pressure greater than 140/90 mmHg on at least two independent readings, exclude secondary hypertension. Diagnosis of ischemic heart disease according to the WHO diagnostic criteria [6]. Diagnosis of diabetes mellitus according to the WHO diagnostic criteria [7], exclude type 1 diabetes mellitus and secondary diabetes.

### Genotyping

Blood samples were collected in tubes containing EDTA. The genomic DNA was extracted using a commercially available DNA isolation kit (Cwbiotech, Beijing, China) according to the manufacturer’s instruction.

The primers of CYP2C19*2 and CYP2C19*3 alleles were designed and synthesized by BGI Beijing Corporation. The forward primer of 5′- ACC AGA GCT TGG CAT ATT GTA TCT -3′ and the reverse primer of 5′-GAT TCT TGG TGT TCT TTT ACT TTC T-3′ were used for the amplification of the CYP2C19*2 allele. For the CYP2C19*3 variant, the forward primer was 5′- TTT CAT CCT GGG CTG TGC TC -3′ and the reverse primer was 5′- TGT ACT TCA GGG CTT GGT CAA T -3′. The PCR was performed with an initial denaturation at 94°C for 5 min, followed by 35 cycles of denaturation at 94°C for 30 s, annealing at 60°C for 30 s, elongation at 72°C for 30 s, and a final extension at 72°C for 10 min. The amplified products of CYP2C19*2 (192bp) and CYP2C19*3 (234bp) were respectively digested with SmaI and BamHI fast-digest restriction enzyme (Takara biotechnology, Dalian).

Samples from each genotype of CYP2C19 were selected randomly and submitted for direct DNA sequencing which confirmed the results.

### Statistical analysis

Continuous variables were expressed as mean ± standard deviation (SD) and categorical variables were reported as counts and percentages. Analyses of t-tests and chi-square tests were used to test for differences between groups for continuous and categorical variables, respectively. Multivariate logistic regression analysis was used to identify independent predictors of cerebral ischemic stroke. Analyses were performed using SPSS version 19.0 statistical software. A value of P < 0.05 (two-sided) was considered statistically significant. The p value is accurate to three decimal places when we calculated using SPSS.

## Results

A total of 299 patients were enrolled in this study, including 177 cases of male, 122 cases of female, the first onset group were 185 cases, the recurrent stroke group were 114 cases, the mean ages were (67.86 ± 11.472) years. And a total of 295 controls were enrolled, including 159 males, 136 females, the mean ages were (66.45 ± 12.972) years.

### Genotyping

**Figure 1:**
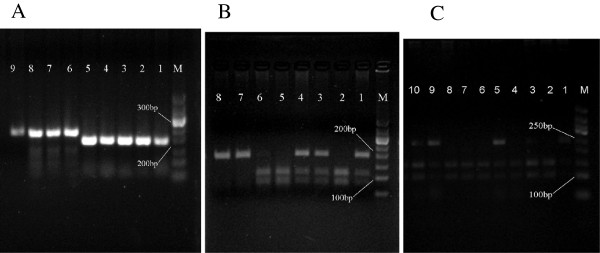
The electrophoresis results of the PCR products and digested products of CYP2C19*2 and CYP2C19*3. A: The PCR products of CYP2C19*2 and CYP2C19*3. Lane M: DNA Marker (50bp~500bp), Lane 1~5: CYP2C19*2, Lane 6~9:CYP2C19*3. **B**: Digested products of CYP2C19*2. Lane M: DNA Marker (50bp~500bp), Lane 1,3,4: heterozygous, Lane 2,5,6: wild-type, Lane 7,8: homozygous. **C**: Digested products of CYP2C19*3. Lane M: DNA Marker (50bp~500bp), Lane 1: homozygous, Lane 2,4,6,7,8: wild-type, Lane 3,5,9,10: heterozygous.

**Figure 2:**
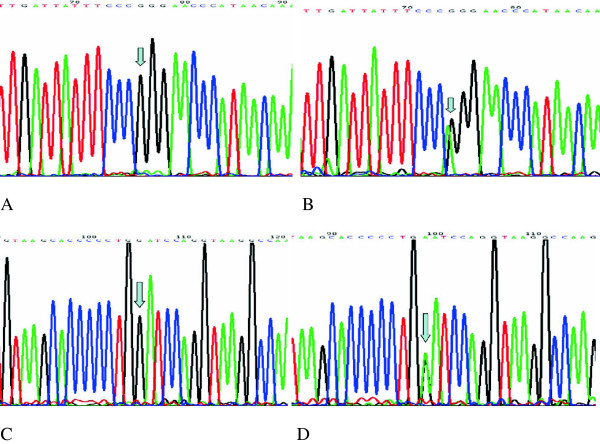
Genetic sequencing maps of CYP2C19*2 and CYP2C19*3. The arrows were pointing to the genetic single nucleotide polymorphisms of CYP2C19. Fig. **A**: wild-type of CYP2C19*2, Fig. **B**: heterozygous of CYP2C19*2, Fig. **C**: wild-type of CYP2C19*3, Fig. **D**: heterozygous of CYP2C19*3.

The PCR products of CYP2C19*2 and CYP2C19*3 were respectively 192bp and 234bp, which were shown in Figure 1A, and their digested products were respectively showed in Figure 1B and Figure 1C.As shown in Figure 2, the results of direct DNA sequencing verified the correctness of genotyping results.

### Clinical characteristics

Baseline clinical characteristics of the cerebral ischemic stroke group and the control group are shown in Table 1. The proportions of hypertension, diabetes, ischemic heart disease, hyperlipidemia, smoking and drinking in the cerebral ischemic stroke group were more than those in the healthy control group. And the levels of fasting blood-glucose (FBG), systolic blood pressure and diastolic blood pressure were significantly higher in the cerebral ischemic stroke than in the control group, while the level of high-density lipoprotein cholesterol (HDL-C) was significantly lower in the cerebral ischemic stroke than in the control group. As shown in Table 1, there were also no significant differences in the levels of total cholesterol (TC), triglyceride (TG) and LDL-C.

**Table 1:** Demographic and clinical information of the cerebral ischemic stroke and the control group

**Variable**	**The cerebral ischemic stroke (n = 299)**	**The control group (n = 295)**	**t/χ2 value**	***P *** **value**
Age (years)	67.86 ± 11.472	66.45 ± 12.972	1.381	0.168
Male (n, %)	177 (59.2)	159 (53.9)	1.697	0.193
Hypertension (n, %)	225 (75.3)	75 (25.4)	147.483	0.000
Diabetes (n, %)	103 (34.4)	21 (7.1)	67.147	0.000
Hyperlipidemia (n, %)	161 (53.8)	122 (41.4)	9.287	0.002
Ischemic heart disease (n, %)	106 (35.5)	36 (12.2)	44.119	0.000
Smoking (n, %)	119 (39.8)	31 (10.5)	67.495	0.000
Drinking (n, %)	72 (24.1)	20 (6.8)	33.956	0.000
Systolic blood pressure (mmHg)	153.01 ± 22.593	127.82 ± 18.146	14.971	0.000
Diastolic blood pressure (mmHg)	86.21 ± 12.644	76.51 ± 9.684	10.489	0.000
Total cholesterol (mmol/L)	5.033 ± 1.366	5.211 ± 1.030	-1.791	0.074
Triglyceride (mmol/L)	1.654 ± 1.116	1.537 ± 1.038	1.322	0.187
High-density lipoprotein cholesterol (mmol/L)	1.113 ± 0.562	1.316 ± 0.452	-4.842	0.000
Low--density lipoprotein cholesterol (mmol/L)	3.157 ± 1.061	3.078 ± 0.815	1.028	0.304
Fasting blood-glucose (mmol/L)	7.504 ± 3.461	5.538 ± 0.980	9.391	0.000

Continuous variables were expressed as mean ± standard deviation (SD) and categorical variables were reported as counts and percentages. The blood pressure is measured in millimetres of mercury (mm Hg). The blood lipids and glucose concentrations are record in milimolar per litre (mmol/L).

Meanwhile, there were no significant differences in the proportions of age, gender, smoking, drinking, hypertension, diabetes, ischemic heart disease, myocardial infarction and hyperlipidemia in the recurrent stroke group and the first onset group. There were no significant differences in the levels of baseline blood pressure, TC, TG, high-density lipoprotein cholesterol (HDL-C) and FBG in the two groups, which were shown in Table 2.

**Table 2:** Demographic and clinical information of the recurrent stroke group and the first onset group

**Variable**	**The recurrent stroke group (n = 114)**	**The first onset group (n = 185)**	**t/χ2 value**	***P *** **value**
Age (years, mean ± SD)	69.37 ± 11.132	68.28 ± 11.149	0.801	0.424
Male (n, %)	62(54.4)	115(62.2)	1.766	0.184
Hyperlipidemia (n, %)	87(76.3)	138(74.6)	0.180	0.672
Diabetes (n, %)	42(36.8)	61(33.0)	0.468	0.494
Hyperlipidemia (n, %)	65(57.0)	96(51.9)	0.746	0.388
Ischemic heart disease (n, %)	48(42.1)	58(31.4)	3.565	0.059
myocardial infarction (n, %)	4(3.5)	12(6.5)	1.235	0.266
Smoking (n, %)	40(35.1)	79(42.7)	1.707	0.191
Drinking (n, %)	23(20.2)	49(26.5)	1.537	0.215
Systolic blood pressure (mmHg, mean ± SD)	150.99 ± 23.368	154.26 ± 22.074	-1.216	0.225
Diastolic blood pressure (mmHg, mean ± SD)	85.82 ± 11.937	86.46 ± 13.086	-0.427	0.670
Total cholesterol (mmol/L, mean ± SD)	4.990 ± 1.619	5.059 ± 1.188	-0.427	0.670
Triglyceride (mmol/L, mean ± SD)	1.567 ± 0.938	1.708 ± 1.212	-1.062	0.289
High-density lipoprotein cholesterol (mmol/L, mean ± SD)	1.056 ± 0.303	1.148 ± 0.672	-1.386	0.167
Low--density lipoprotein cholesterol (mmol/L, mean ± SD)	3.148 ± 1.325	3.163 ± 0. 862	-0.119	0.905
Fasting blood-glucose (mmol/L, mean ± SD)	7.733 ± 3.798	7.363 ± 3.239	0.898	0.370

### Frequency of CYP2C19 genotypes and alleles

The frequency of the major and minor alleles of CYP2C19*2 and CYP2C19*3 did not deviate significantly from the Hardy-Weinberg equilibrium neither in the stroke (χ2 = 1.380, *P* = 0.502; χ2 = 4.180, *P* = 0.124), nor in the control group (χ2 = 1.749, *P* = 0.417; χ2 = 0.400, *P* = 0.819). Frequencies of the CYP2C19*2 and CYP2C19*3 polymorphism in cerebral ischemic stroke patients and healthy controls are described in Table 3. Frequencies of CYP2C19 681AA (30.9% vs. 20.8%; *P* = 0.000), CYP2C19 636AA (4.0% vs. 0.7%; *P* = 0.007), CYP2C19 636AG (18.1% vs. 10.2%; *P* = 0.006) genotype, CYP2C19 681A (30.9% vs. 20.8%; *P* = 0.000) and CYP2C19 636A (13.0% vs. 5.8%; *P* = 0.000) allele were significantly higher in the cerebral ischemic stroke than in the control group.

**Table 3:** Frequencies of the CYP2C19 polymorphism in cerebral ischemic stroke patients and healthy controls (n, %)

**Groups**	CYP2C19*2 genotypes			CYP2C19*2 alleles		CYP2C19*3 genotypes			CYP2C19*3 alleles	
	**AA**	**AG**	**GG**	**A**	**G**	**AA**	**AG**	**GG**	**A**	**G**
Stroke	35	115	149	185	413	12	54	233	78	520
(n = 299)	(11.7)	(38.5)	(49.8)	(30.9)	(69.1)	(4.0)	(18.1)	(77.9)	(13.0)	(87.0)
Control	8	107	180	123	467	2	30	263	34	556
(n = 295)	(2.7)	(36.3)	(61.0)	(20.8)	(79.2)	(0.7)	(10.2)	(89.2)	(5.8)	(94.2)
χ2 value	17.887	0.304	7.517	15.741		7.178	7.615	13.584	18.437	
*P* value	0.000	0.581	0.006	0.000		0.007	0.006	0.000	0.000	

Frequencies of the CYP2C19*2 and CYP2C19*3 polymorphisms in recurrent stroke group and the first onset group are described in Table 4. Frequencies of CYP2C19 681AA (16.7% vs. 8.6%; *P* = 0.036), CYP2C19 636AA (7.0% vs. 2.2%; *P* = 0.038) genotype, CYP2C19 681A (36.4% vs. 27.6%; *P* = 0.023) and CYP2C19 636A (17.5% vs. 10.3%; *P* = 0.010) allele were significantly higher in recurrent stroke group than in the first onset group.

**Table 4:** Frequencies of the CYP2C19 polymorphism in recurrent stroke group and the first onset group (n, %)

**Groups**	CYP2C19*2 genotypes			CYP2C19*2 alleles		CYP2C19*3 genotypes			CYP2C19*3 alleles	
	**AA**	**AG**	**GG**	**A**	**G**	**AA**	**AG**	**GG**	**A**	**G**
The recurrent stroke group	19	45	50	83	145	8	24	82	40	188
(n = 114)	(16.7)	(39.5)	(43.9)	(36.4)	(63.6)	(7.0)	(21.1)	(71.9)	(17.5)	(82.5)
The first onset group	16	70	99	102	268	4	30	151	38	332
(n = 185)	(8.6)	(37.8)	(53.5)	(27.6)	(72.4)	(2.2)	(16.2)	(81.6)	(10.3)	(89.7)
χ2 value	4.387	0.080	2.629	5.155		4.316	1.115	3.852	6.580	
*P* value	0.036	0.778	0.105	0.023		0.038	0.291	0.050	0.010	

Neurological functions were evaluated immediately with the US National Institutes of Health nerve function deficit score (NIHSS) after admission to hospital. Patients were divided into mild (NIHSS<5), moderate (5 ≤ NIHSS ≤ 20) and severe (NIHSS>20) by rating results [8]. As shown in Table 5, no significant differences were found in genotype (χ2 = 4.967, *P* = 0.291; χ2 = 3.239, *P* = 0.519) and allele (χ2 = 0.616, *P* = 0.735; χ2 = 1.242, *P* = 0.537) frequencies of CYP2C19*2 and CYP2C19*3 in the three groups.

**Table 5:** Frequencies of the CYP2C19 polymorphism in different NIHSS groups in patients

**Groups**	CYP2C19*2 genotypes			CYP2C19*2 alleles		CYP2C19*3 genotypes			CYP2C19*3 alleles	
	**AA**	**AG**	**GG**	**A**		**AA**	**AG**	**GG**	**A**	**G**
Mild	19	64	78	102	220	6	34	121	46	276
(n = 161)	(11.8)	(39.8)	(48.4)	(31.7)	(68.3)	(3.7)	(21.1)	(75.2)	(14.3)	(85.7)
Moderate	8	37	39	53	115	4	10	70	18	150
(n = 84)	(9.5)	(44.0)	(46.4)	(31.5)	(68.5)	(4.8)	(11.9)	(83.3)	(10.7)	(89.3)
Severe	8	14	32	30	78	2	10	42	14	94
(n = 54)	(14.8)	(25.9)	(59.3)	(27.8)	(72.2)	(3.7)	(18.5)	(77.8)	(13.0)	(87.0)
χ2 value	0.893	4.806	2.432	0.616		0.170	3.176	2.147	1.242	
*P* value	0.640	0.090	0.296	0.735		0.919	0.204	0.342	0.537	

### Correlation between CYP2C19 genetic polymorphisms and cerebral ischemic stroke

Multivariate logistic regression analysis showed that the CYP2C19 681AA genotype was an independent risk factor for cerebral ischemic stroke (odds ratio (OR) 6.179, 95% confidence interval (CI) 2.285 ~ 16.708; *P* = 0.000), which were shown in Table 6, and the CYP2C19 681 AA genotype was an independent risk factor for recurrent stroke (OR 2.305, 95% CI 1.121 ~ 4.743; *P* = 0.023), while CYP2C19 636 AA genotype was not associated with the recurrence of ischemic stroke (*P* = 0.098). Meanwhile, as shown in Table 6, hypertension was an independent risk factor for cerebral ischemic stroke (OR 2.998, 95% CI 1.808 ~ 4.972; *P* = 0.000).

**Table 6:** Multivariate logistic regression analysis of independent risk factor for cerebral ischemic stroke

**Independent risk factor**	**95% CI**	**P value**	**OR value**
CYP2C19 681AA	2.285~16.708	0.000	6.179
Hypertension	1.808~4.972	0.000	2.998
Ischemic heart disease	1.366~4.210	0.002	2.398
Diabetes	1.262~4.838	0.008	2.471
Hyperlipidaemia	1.086~3.120	0.023	1.841
Smoking	3.712~11.816	0.000	6.623
Systolic blood pressure	1.036~1.065	0.000	1.051
TC	0.626~0.924	0.006	0.761
FBG	1.257~1.742	0.000	1.480

## Discussion

CYP2C19 is located within a cluster of cytochrome P450 genes on chromosome 10q24, which contains nine exons and eight introns. The gene encodes a 490-aa long protein of approximately 56kDa, which is a member of the cytochrome P450 superfamily of enzymes. This protein localizes to the endoplasmic reticulum and is known to metabolize many xenobiotics, including the anticonvulsive drug mephenytoin, omeprazole, diazepam and some barbiturates. Polymorphism within this gene is associated with variable ability to metabolize mephenytoin, known as the poor metabolizer and extensive metabolizer phenotypes. CYP2C19*2 has been shown to be a G → A transition at 681bp in exon 5 of wild-type CYP2C19*1. This variant results in an aberrant splice site and shifts the reading frame, thereby producing an early-stop codon and a truncated protein [9]. The CYP2C19*3 involves a G → A variant at 636bp in exon 4, that also creates a premature stop codon and a truncated protein [10]. The CYP2C19*2 is the main genetic defect allele of CYP2C19, which accounts for 75% ~ 85% of poor metabolizers (PM) in both white and Oriental populations [11]. The second variant accounts for the remaining defective alleles in Oriental populations PM, but appears to be extremely rare in white persons [12].

Yin *et al*. [13] found that the allele frequencies of CYP2C19*2 were respectively 29.7%, 32.4% and 18.2% in Han, Hui and Mongolian, the allele frequencies of CYP2C19*3 were respectively 7.9%, 10.2% and 11.2% in Han, Hui and Mongolian. Other literatures [14,15] reported that the allele frequencies of CYP2C19*2 were 11.1% for Western Africa, 14.0% for Western Europe and 16.3% for Northern Europe respectively, and the allele frequencies of CYP2C19*3 were 0% for Western Africa, 0.2% for Western Europe and 0.2% for Northern Europe respectively. In our study, the allele frequencies of CYP2C19*2 and CYP2C19*3 were 25.9% and 9.4% separately. Our result is close with that in Yin’s report.

Studies have shown that the poor metabolizer gene of CYP2C19 was related to the occurrence of coronary heart disease and stroke. Through the research of 654 patients with coronary heart disease, Mao Chen *et al*. [16] found that the homozygous CYP2C19*2/*2 genotype was an independent determinant of adverse vascular events in Chinese patients with coronary artery disease (CAD). Mega *et al*. [17] reported that carriers of a reduced-function CYP2C19 allele had a higher rate of major adverse cardiovascular events than did noncarriers. Actually our research also found that CYP2C19 681AA genotype, CYP2C19 636AA and CYP2C19 636AG genotype were related with cerebral ischemic stroke. Multivariate logistic regression analysis showed that the CYP2C19 681AA genotype may be an independent risk factor for cerebral ischemic stroke. We postulate that the weak metabolic gene of CYP2C19 may be related to the formation of cerebral artery atherosclerosis, thereby causing the incidence of cerebral ischemic stroke.

Clopidogrel is a prodrug requiring metabolism by CYP2C19 enzyme, in order to be active, which can play the role of anti-platelet aggregation. Clopidogrel is widely used in the secondary prevention of cerebral ischemic stroke, but its clinical efficacy has individual differences. Many studies have shown that CYP2C19 polymorphism is associated with reduced clopidogrel response [18-23]. Our study suggested that the CYP2C19 681AA genotype was an independent risk factor for recurrent stroke, since carriers of a reduced-function CYP2C19 allele had a two-fold risk with recurrent stroke than did noncarriers.

## Conclusion

In summary, the AA genotype and A allele of CYP2C19 G681A may be associated with the occurrence and recurrence of cerebral ischemic stroke. In addition, we find that the genotypes of CYP2C19 G681A and G636A have nothing to do with the severity of cerebral ischemic stroke. Since our study has limited sample size, further studies in a large population are needed to confirm these findings.

## Abbreviations

CYP: Cytochrome P450; CIS: Cerebral ischemic stroke; SNPs: Single nucleotide polymorphisms; CT: Computed tomographic; MRI: Magnetic resonance imaging; TIA: Transient ischemic attack; SD: Standard deviation; FBG: Fasting blood-glucose; HDL-C: High-density lipoprotein cholesterol; TC: Total cholesterol; TG: Triglyceride; OR: Odds ratio; CI: Confidence interval.

## Competing interests

The authors declare that they have no competing interests.

## Authors’ contributions

LX applied the grants and designed the proposal. GS carried out the laboratory work, analyzed the data and drafted the manuscript. SY guided the molecular biology experiments and revised the manuscript. Before submission each author have read and given final approval of the manuscript. All authors read and approved the final manuscript.

## Pre-publication history

The pre-publication history for this paper can be accessed here:

http://www.biomedcentral.com/1471-2350/15/83/prepub
